# Isolation and Characterization of Some Phytochemicals from Indian Traditional Plants

**DOI:** 10.1155/2012/549850

**Published:** 2012-12-11

**Authors:** Neeharika Srivastava, Aishwarya Singh Chauhan, Bechan Sharma

**Affiliations:** Department of Biochemistry, Faculty of Science, University of Allahabad, Allahabad 211002, India

## Abstract

The present study was designed to evaluate relative contribution of different polyphenols (total phenolics, flavonoids, flavonols) and their antioxidants activities in aqueous extracts of different parts of some plants; *Argemone mexicana*, *Datura metel*, *Calotropis procera*, *Thevetia peruviana*, and *Cannabis sativa*. The antioxidants (total phenolics, flavonoids, flavones) were determined by chemical methods. The antioxidant capacities of these extracts were evaluated by FRAP assay. The results demonstrated that phenolic content was maximally present in leaves of *T. peruviana*. This plant exhibited minimum phenolic content in its flower as compared to other plants. The flower of *D. metel* contained maximum phenolic content. The flavonoids were present in highest quantity in leaves of *C. procera* while *T. peruviana* flowers showed maximum flavonoid content. The fruits of *C. sativa* contained maximum quantity of flavonoid as compared to other plants tested. The flower extract of *C. sativa* possessed highest FRAP value followed by *A. mexicana* and fruit of *C. procera.* The values of ratios of different polyphenolic compounds present in plant extracts indicated that flower of *D. metel* contained maximum total flavonoids and minimum phenolics. These results suggested that levels of total phenolics, flavonoids and their FRAP indices exhibited specificity to different plants and their parts.

## 1. Introduction

The extraction of plant constituents is essential to isolate biologically active compounds and in understanding their role in disease prevention and treatment and in knowing their toxic effects as well. However, meager information is available about the medicinal and pharmacological properties and biological activities of phytochemicals derived from some plants (*Calotropis procera*, *Datura metal*, *Cannabis sativa*, *Argemone Mexicana,* and *Thevetia peruviana*) commonly known to have toxic, narcotic and ornamental properties. The information available on these important plants indicates that not much attention has been paid towards studying their physicochemical properties as well as biological activities towards their potentials as antioxidants. Keeping this information in view, an endeavour has been made in this communication to determine some biochemical constituents and their properties into the aqueous extracts of aforesaid medicinally important plants commonly available in the northern part of India. 

*Calotropis procera*, known as apple of Sodom or mudar, belongs to Apocynaceae family and is found in many countries such as Africa and Western and South Asia, as well as Indochina. It is known for its medicinal and pharmacological properties [1]. The milky sap of this plant is known to contain three toxic glycosides: (i) calotropin, (ii) uscharin, and (iii) calotoxin as well as steroidal heart poisons, known as cardiac aglycones [2]. The crude extract of this plant and its protein fraction possess high fibrinolytic and anticoagulant activity in rabbit and human plasma [3]. Aqueous extracts of different parts of this plants are shown to exert mild diuretic and cardiac as well as respiratory stimulating effects in experimental animals [4].

*Datura metal*, a well-known traditional Indian plant, is found throughout the warmer parts of the world and contains both the ornamental and medicinal properties. All parts of *Datura* plants contain high levels of tropane alkaloids, which are highly toxic to humans and other animals. This plant is known to possess analgesic [5, 6], antioxidant, and antimicrobial properties [7].

*Argemone mexicana*, known as Mexican poppy orMexican prickly poppy, belongs to the species of poppyfound inMexicoand nowin theUnited States,India,andEthiopia. It is poisonous but has been used as a traditional medicine bynativesof the western US and parts ofMexico [8]. It possesses the alkaloid sanguinarine reported to be responsible for epidemic dropsy [9, 10]. *A. mexicana* is reported to have antimicrobial activity [11], wound healing capacity in rat [12], larvicidal and chemosterilant activity [13], and nematicidal and allelopathic potential [14].

The root of *Cannabis sativa* is used as an old folk medicine to treat arthritis or joint pain [15]. *C. sativa* contains tetrahydrocannabinol (THC), which is of medical significance [16]. The cannabidiol isolated from *C. sativa* may act as an antipsychotic drug [17]. Terpenes have been detected and isolated from essential oil from flowers, leaves, and roots [18]. The terpenes are responsible for the flavor of different varieties of cannabis [19]. The roots, leaves, stems, pollen, and seeds of *C. sativa* contain piperidine and pyrrolidine, which have been used in different medicinal formulations [20].

*Thevetia peruviana*, a native in Iran, the Mediterranean regions, and India, produces milky juice. The leaves of this plant are used as a cardiotonic, antibacterial, and diuretic agent and used also to treat cutaneous eruptions and as an antidote against snake venom [21]. Its root is used for curing different types of cancers, ulcers, and leprosy while the root bark is used specifically against ring worm. The aqueous extracts of its leaves, branches, roots, and flowers are toxic to certain insects [22]. The main phytochemicals found in different parts of this plant include glycosides, terpenoids, cardiotonic substances, and steroids.

Oxidation and reduction of molecules are the common reactions in every cell leading to the production of free radicals and these free radicals react with organic substrates, namely, lipids, proteins, and DNA, causing damage to these molecules. Due to altered redox homoeostasis, an imbalance between the production and neutralization of the free radicals within the cell gives rise to oxidative stress (OS). It disturbs their normal functions resulting in the onset of variety of chronic diseases including cancer, heart disease, and other degenerative diseases. A wide variety of sources are present in biological systems producing reactive free radicals and reactive oxygen species. Considering the pathomechanism of free radicals, certain diseases are named as free radical diseases [23]. The organ systems most susceptible to damage are the eyes, brain, pulmonary, circulatory, and the reproductive systems [24–27].

The presence of the excess of oxidants (free radicals and nonradical reactive molecules) derived from free radicals such as reactive oxygen species (ROS): hydroxyl (OH^•^), superoxide (O_2_^•−^), nitric oxide (NO^•^), thiyl (RS^•^), and peroxyl (RO^•^_2_)is known to cause oxidative stress. The nonradical ROS includes peroxynitrite (ONOO^−^), hypochlorous acid (HOCl), hydrogen peroxide (H_2_O_2_), singlet oxygen (–^1^O_2_), ozone (O_3_), and lipid peroxide (LOOH) as well as reactive nitrogen species (RNS) such as nitrous oxide (N_2_O), nitrosyl cation (NO^+^), peroxynitrite (OONO^−^), nitrogen dioxide (NO^•^_2_), peroxynitrous acid (ONOOH), dinitrogen trioxide (N_2_O_3_), nitroxyl anion (NO^−^), nitrous acid (HNO_2_), and nitryl chloride (NO_2_Cl) [28, 29]. 

There are evidences which suggest that by quenching the free radicals, antioxidants help reduce the risk of chronic diseases. These antioxidants are either endogenous (internally synthesized) or exogenous (consumed). Nowadays, the application of plant based antioxidants or natural antioxidants is replacing synthetic molecules because of toxicities associated with the later [30, 31]. The phytochemicals like phenolic acids, polyphenols, flavonoids, flavonols, terpenoids vitamin C, vitamin E, carotenes, phenolic acids, phytate, and phytoestrogens scavenge the free radicals activity thus inhibiting the oxidative mechanisms that lead to emergence of various diseases as these molecules are electron rich. They donate electrons to ROS and neutralize these chemical species [32, 33].

In this study we have selected the aforesaid five plants, namely, *Calotropis procera*, *Datura metal*, *Cannabis sativa*, *Argemone Mexicana,* and *Thevetia peruviana* known for their varied properties. *C. procera *has the medicinal and toxic constituents, *D. metal* contains narcotics and toxic substances, *C. sativa* possesses some narcotics, *A. mexicana *causes dropsy, and *T. peruviana* has medicinal and ornamental applications. In our laboratory, some of the properties of the phytochemicals present in aqueous extracts of different parts (leaves, stem, flowers, and fruits) of these plants have been explored. In this paper, the presence of polyphenolic contents (total phenolics, flavonoids, and flavonols) in these aqueous extracts as well as their antioxidant potentials have been demonstrated. 

## 2. Materials and Methods

### 2.1. Chemicals

2,4,6-tripyridyl-*s*-triazine (TPTZ), ferrous sulphate, AlCl_3_, and FeCl_3_ were purchased from Sisco Research Laboratory; quercetin was purchased from Sigma Chemical Co. (St. Louis, MO, USA); Folin-Ciocalteu's phenol reagent and sodium carbonate were from Merck Chemical Supplies (Darmstadt, Germany). All the other chemicals used including the solvents were of analytical grade.

### 2.2. Collection and Identification of Plant Materials

Different parts (leaves, stem, flower, and fruits) of some plants such as *Argemone mexicana, Datura metal*, *Calotropis procera*, *Thevetia peruviana,* and* Cannabis sativa* were used in this study. These plant samples were collected from Allahabad and adjoining areas during March and April (38 ± 1°C) in the year 2011. 

### 2.3. Preparation of Plant Extracts

The fresh plant parts were collected washed with tap water followed by distilled water. 5.0 g of each was cut into several small pieces, minced well in a pestle and a mortar, and extracted with 50 mL of 50 mM Tris-HCl buffer at (pH 7.0). Freezing and thawing are done twice at the intervals of 2 h each followed by mechanical jerk by grinding in the pestle mortar in order to rupture the plant cell wall. The 10% (w/v) homogenate of each of the plant materials was prepared at 4–6°C. The homogenate was filtered using Whatman's filter paper type 1. The volume of the filtrate was recorded. The filtrate was centrifuged at 1000 xg for 10 min under cooling (4–6°C) conditions. The clear supernatant was used to estimate their antioxidant potential. The difference of the weights of the starting material and the residues was considered as the amount of the plant present in the extract.

### 2.4. Determination of Total Phenolics

Folin-Ciocalteu method as described elsewhere [34] was employed for estimation of total phenolics in the aqueous plant extracts. An aliquot (100 *μ*L) of the extracts was mixed with 2.5 mL Folin-Ciocalteu reagent (previously diluted with water; 1 : 10 v/v) and 2 mL (75 g/L) of sodium carbonate. The tubes were vortexed for 15 s and allowed to stand for 30 min at 40°C for color development. The optical absorbance was recorded against reagent blank at 765 nm wavelength using the Thermoscientific Spectrascan UV2700 double beam spectrophotometer. The concentration of each plant extract was 0.1 g/mL. Total phenolic contents were expressed as mg/g n-propyl gallate equivalent.

### 2.5. Determination of Total Flavonoids

Determination of total flavonoid content was done using the method already described elsewhere [35]. In brief, a volume of 0.5 mL of 2% AlCl_3_ in ethanol solution was added to 0.5 mL of plant extracts. After 1 h incubation at room temperature, the absorbance was measured at 420 nm. Appearance of yellow color indicated the presence of flavonoids. The extract samples were evaluated at a final concentration of 0.1 g/mL. Total flavonoid contents were calculated as quercetin equivalent (mg/g).

### 2.6. Determination of Total Flavonols

Total flavonols in the plant extracts were estimated by a known method described elsewhere [36]. In brief, 1.0 mL of 2% AlCl_3_ in ethanol and 1.5 mL sodium acetate (50 g/L) solutions were added in 0.10 mL of extract solution. The absorption at 440 nm was monitored after 2.5 h of incubation at 20°C. The sample extracts were evaluated at a final concentration of 0.1 mg/mL. Total flavonoid content was calculated as quercetin equivalent (mg/g).

### 2.7. Total Antioxidant Activity Determination by Ferric Reducing Antioxidant Power (FRAP) Assay

The method as described by Benzie and Strain [37] with some modifications was employed for the estimation of antioxidant activity by FRAP assay. The stock solutions included 300 mM acetate buffer (pH 3.6), 10 mM 2,4,6-tripyridyl-*s*-triazine (TPTZ) solution in 40 mM HCl, and 1 mM FeCl_3_ · 6H_2_O solution. TPTZ was dissolved in 40 mM HCl at 50°C in water bath for 30–40 min till it completely dissolves. The fresh working solution was prepared by mixing 10 : 1 : 1 of acetate buffer, TPTZ, and FeCl_3_ · 6H_2_O, respectively. The temperature of the working solution was maintained to 37°C before starting the reaction by adding the plant extracts (100 *μ*L) to 2 mL of the FRAP solution. The reaction mixture was incubated for 30 min in the dark condition. The optical absorbance of the colored product (ferrous tripyridyltriazine complex) was recorded at 593 nm. The standard curve was linear between 20 and 100 *μ*M FeSO_4_ · 7H_2_O. The results were expressed in *μ*M Fe (II)/g dry mass.

## 3. Results

### 3.1. The Evaluation of Phenolics in the Aqueous Extracts of Different Parts of the Plants

**Figure 1:**
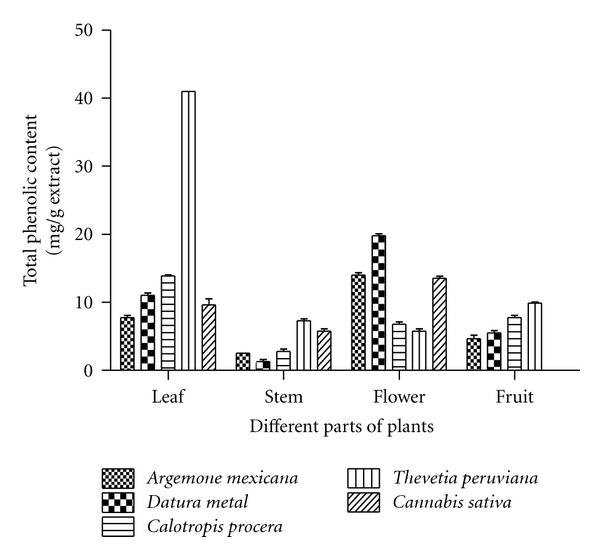
Comparative estimates of the analysis of total phenolic content in different parts of *A. mexiacana, D. metal, C. procera, T. peruviana, *and* C. sativa.* The determination of phenolics has been done as described in Section 2. The results indicate average values of three independent experiments.

The data obtained after analysis of total phenolics as shown in Figure 1 was largely variable not only among the plants but also among their various parts. In case of *A. mexicana,* the highest phenolic content was found in flowers (14 mg/g) followed by leaf, fruit, and stem, with the values being 7.5, 4.62, and 2.5 mg/g, respectively. The aqueous extracts of *D. metal* showed a similar pattern with maximum phenolic content present in flowers (19.75 mg/gm) followed by leaf, fruit, and stem, with the values being 11, 5.5, and 1.25 mg/g, respectively. In *C. procera,* maximum phenolic content was present in leaves (14 mg/g). Other parts of *C. procera* such as fruit, flower, and stem, had values of 7.7, 6.7, and 2.7 mg/g, respectively. Among the different parts of *T. peruviana,* maximum phenolic content was found in leaves (41 mg/g) followed by fruit, stem, and flower, with the values being 9.75, 7.3, and 5.75 mg/g, respectively. The flower of *C. sativa* contained maximum phenolic content (13.5 mg/g) followed by leaf and stem, with the values being 9.62 and 5.7 mg/g, respectively (Figure 1). 

Upon comparison of all the five plants, the leaves of *T. peruviana* were found to have maximum phenolic content followed by leaves of *C. procera*, *D. metal*, *C. sativa,* and *A. mexicana*. Stems of all the plants did not contain a significant quantity of phenolics (Figure 1). In case of flowers, *D. metal* contained maximum phenolic content followed by that of *A. mexicana*, *C. sativa*, *C. procera,* and *T. peruviana. *The trend of total phenolic content in different parts of the selected plants is as the following: leaves: *T. peruviana > C. procera > D. metal > C. sativa > A. mexicana;* stem: *T. peruviana > C. sativa > C. procera > A. mexicana > D. metal; *flower: *D. metal > A. mexicana > C. sativa > C. procera > T. peruviana* and: fruit: *T. peruviana > C. procera > D. metal > A. mexicana *(Figure 1).

### 3.2. The Analysis of Flavonoids in the Aqueous Extracts of Different Parts of the Plants

**Figure 2:**
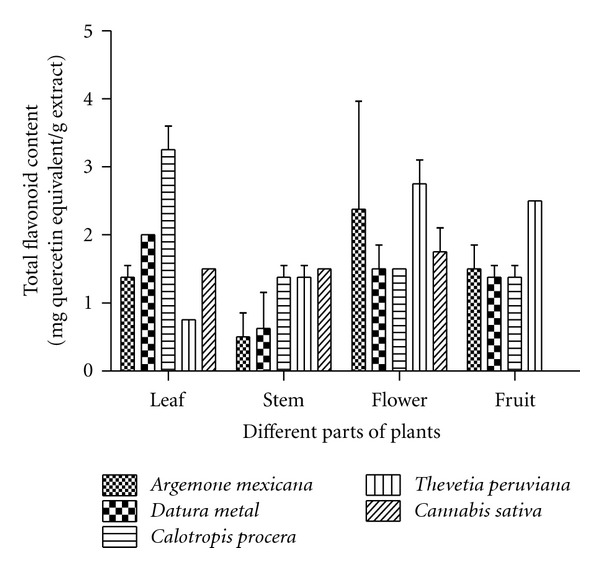
Comparative estimates of the analysis of total flavonoid content in different parts of *A. mexiacana, D. *metal*, C. procera, T. peruviana, *and* C. sativa.* The determination of flavonoids has been done as described in Section 2. The results indicate average values of three independent experiments.

Like the phenolics in the aqueous extracts of aforesaid plants, the analysis of flavonoid was also carried out. As shown in Figure 2, different parts of the plants exhibited the presence of flavonoids, albeit to varying extents. In *A. mexicana,* the highest flavonoid content was found in flowers (2.37 mg/g) followed by fruit, leaf, and stem, with the values being 1.5, 1.37, and 0.5 mg/g, respectively. In *D. metal,* the highest flavonoid content was found in leaves (2 mg/g) followed by flower, fruit, and stem, with the values being 1.5, 1.37, and 0.62 mg/g, respectively. The *C. procera* leaves exhibited highest flavonoid content (3.25 mg/g) whereas its stem, flower, and fruit contained 1.25, 1.5, and 1.75 mg/g flavonoid, respectively. The *T. peruviana,* flowers and fruits were having nearly the same level of flavonoid contents (2.6 and 2.5 mg/g) whereas its stem and leaves contained 1.37 and 0.75 mg/g flavonoid, respectively. The flowers of *C. saiva *displayed the presence of highest flavonoid content (1.75 mg/g). Its leaves and stem had almost the same amount (1.5 mg/g) of flavonoid (Figure 2). 

An organwise comparison of the five plants suggested that the maximum flavonoid content was found in the leaves of *C. procera* followed by *D. metal, C. sativa, A. Mexicana,* and *T. peruviana*. In the stem, *C. procera, T. peruviana,* and *C. sativa* had almost same flavonoid content while *A. mexicana* and *D. metal* contained relatively low level of this molecule. The flowers and fruits of *T. peruviana* were found to contain maximum flavonoid content as compared to other plants tested. The trend of total flavonoid content in different parts of the selected plants is as the following: leaves: *C. procera > D. metal > C. sativa > A. mexicana > T. peruviana; *stem: *C. sativa > C. procera = T. peruviana > D. metal > A. mexicana; *flower: *T. peruviana > A. Mexicana > C. sativa > D. metal = C. procera:* fruit: *T. peruviana > A. mexicana > C. procera > D. metal *(Figure 2).

### 3.3. The Evaluation of Flavons in the Aqueous Extracts of Different Parts of the Plants

The analysis of flavon content in these plants preparations indicated its presence in low quantity in *A. mexicana* only. Other plants tested exhibited absence of flavon in their aqueous extracts (data not shown).

### 3.4. The Evaluation of Ferric Reducing Antioxidant Power (FRAP) in the Aqueous Extracts of Different Parts of the Plants

When these plant extracts were subjected to FRAP assay, flowers of *C. sativa* showed a significant antioxidant potential (74.8 ± 1.93 *μ*M Fe^++^g^−1^) among all the plants. *A. mexicana* flowers, *D. metal* leaves, fruits from *C. procera,* and *T. peruviana* showed maximum antioxidant capacity, with the values being 69.1 ± 0.28, 24.7 ± 1.13, 41.3 ± 1.20, and 24.2 ± 0.31 *μ*M Fe^++^g^−1^, respectively (Table 1).

### 3.5. The Levels of Ratios of Polyphenolic Compounds in the Aqueous Extracts of Different Parts of the Plants

The ratios of polyphenolic compounds present in these plant extracts are shown in Table 2. The data indicated varying levels of flavonoid dependent antioxidant activities in the extracts of different parts of the plants. The trend of presence of flavonoid was found to be as follows: leaves of *A. Mexicana *> flowers of *D. metal *> stem of *C. procera* > flowers of *T. peruviana* > stem of *C. sativa* (Table 2), while among the flowers from all the five plants, *D. metal* exhibited maximum levels and leaves of *T. peruviana *exhibited minimum levels.

## 4. Discussion

It is well known that plant polyphenols, the secondary metabolites, are widely distributed in the plant kingdom and that they are sometimes present in surprisingly high concentrations [38]. Phenolic compounds are characterized by the presence of several phenol groups. By donating a hydrogen atom or an electron they make them very reactive in neutralizing free radicals, chelating metal ions in aqueous solutions [39]. The results of present study indicated that the amount of phenolic contents varied not only plantwise but also from one part of the plant to another. The leaves of *T. peruviana* had maximum phenolic content as compared to the leaves of *C. procera*, *D. metal*, *C. sativa,* and *A. mexicana*, while flowers of *D. metal* contained maximum phenolic as compared to the other plants. Stems of all the plants did not contain any significant quantity of phenolics. The aqueous extract of* Datura stramonium* has been reported to exhibit different levels of phenolic contents in different parts of the plant, with the values in leaf, seed, whole fruit, and stem, respectively, being 0.397, 0.277, 0.1, and 0.114 mg/g [40]. Liu et al. (2008) have shown total phenolic content into Cannabis fruit to be 0.57 ± 0.002 (mg GAE/g dw) [41].

While screening 70 medicinal plant extracts for their antioxidant capacity and total phenols, Katalinic et al. have reported the presence of phenolic contents in different plants to the varying extents [42]. These compounds act as free radical scavengers and thus help protect cells from oxidative toxicity [43–45]. Some workers have demonstrated the presence of phenolic compounds in the aerial parts of the plants including *C. procera, T. peruviana,* and *C. sativa* [46–48] but analysis of these phytoconstituents in the aqueous extracts of different specific parts of these plants has not been worked out. 

It is reported that in the Fenton reaction, flavonoids as antioxidants interfere with the biochemical pathways which are involved in the generation of free radicals (ROS), quench them, chelate the transition metals, and make them redox inactive [49]. Commonly flavonoids occur as glycosides in plants and are considered to be very efficient as antioxidants. With different degrees of hydroxylation, oxidation, and substitution, the flavonoids have common diphenylpropane structure (C_6_C_3_C_6_) [40, 50]. 

The results of the present study reflected that the quantity of flavonoids varies from one plant to another and also into different parts of the plants. Maximum flavonoid content was present in the leaves of *C. procera* and flowers as well as fruits of *T. peruviana *when compared to *D. metal, C. sativa, A. Mexicana,* and *T. peruviana*. The stem of these plants contained low amount of flavonoid. Liu et al. have reported flavonoids content to be absent in Cannabis fruit [41]. Very recently, the levels of flavonoids in different medicinal plants have been reported by de Queiroz Siqueira et al. [51] and they have demonstrated the similar distribution pattern of the flavonoids in specific parts of different plants. According to a hypothesis proposed by Tattini et al. [52], flavonoids have protective functions during drought. Ryan et al. [53] have demonstrated that these molecules impart photoprotection. Likewise, Barceló and Poschenrieder [54] have shown that flavonoids in plants may help ameliorate toxicity of aluminium as they grow in soils contaminated with this heavy metal. Thus in addition to acting as antioxidants, flavonoids are also involved in the regulation of various physiochemical behaviours of plants.

The antioxidant capacity of the plant extract largely depends on both the composition of the extract and the test system [55]. The FRAP assay [37, 56] measures antioxidant power with the help of an oxidant, that is, Fe^3+^. Reduction of ferric to ferrous ion at low pH produces a coloured ferrous-tripyridyltriazine complex. In the FRAP assay, reductants (antioxidants) present in the sample reduce the Fe (III)*/*tripyridyltriazine complex to the blue ferrous form. The change in absorbance and FRAP value of the antioxidants is proportional to each other [56]. The FRAP assay in spite of being simple and inexpensive does have few drawbacks too like the antioxidant capacity of certain antioxidants cannot be measured accurately by this assay such as iron (II) and SH group-containing antioxidants [37, 56–58]. 

The evaluation of FRAP value has been made in the aqueous extracts of different parts of the five plants tested in the present study and the results suggested that flowers of *A. mexicana* and *C. sativa*, *D. metal* leaves, and fruits from *C. procera* and *T. peruviana* exhibited maximum antioxidant capacity. The trend of FRAP values obtained from different plant parts having maximum antioxidant potential for being used in various pharmacological preparations is flowers of *C. sativa* > flowers of *A. mexicana*> fruits of *C. procera *> leaves of *D. metal *> fruits of *T. peruviana*. These results are in agreement with those reported by Katalinic et al. [42]. In another species of Datura, that is, *Datura stramonium*, Oseni et al., (2011) have reported aqueous extracts of leaf, seed, whole fruit, and stem to exhibit 68.90, 25.60, 62.70, and 96.69% antioxidant properties, respectively, by using FRAP assay [40]. Ozgen et al. have presented a study on antioxidant properties of medicinal plants belonging to Asclepiadoideae family, in which *Calotropis gigantea* was reported to have 185.71, 186.13, and 93.07 mmol 100 g^−1^ distilled water, in root, flower, and leaf, respectively, using FRAP assay [57]. Liu et al. have shown FRAP value in Cannabis fruit to be 0.010 ± 0.001 (Fe(II) mmol/g dw) [41].

While evaluating the antioxidant potential of several medicinal plants using FRAP, Katalinic et al. have demonstrated the presence of antioxidant potential of plants to varying degrees. However, the values of FRAP determined by them in their tested medicinal plants were much higher than reported into five different plants under the present study [42, 59]. According to Adedapo et al., the extracts from plants, *Bidens pilosa* and *Chenopodium album,* prepared in acetone and methanol showed relatively high FRAP activity in comparison to the FRAP values obtained using aqueous extracts [60]. Tawaha et al. have recorded a large variation in the total antioxidant capacity of the aqueous and methanolic extracts of the selected Jordanian plant species analyzed [61], which could be attributed to substantial differences in the solubility of phytochemicals extracted into organic and aqueous solvents. The strong correlation observed in the present study between antioxidant activity, phenolics, and flavonoid content of different plants suggests a possible use of their partsin making the active ingredients of antioxidant supplement after removing their toxic ingredients, if any. 

## 5. Conclusion

The results from the present study demonstrated that the leaves of *T. peruviana *contained the presence of the maximum phenolic content. This plant exhibited minimum phenolic content in its flower as compared to others. Phenolic contents were maximum in the flowers of *D. metal*. The flavonoids were present in highest quantity in the leaves of *C. procera* while the *T. peruviana* flowers showed maximum flavonoid content. The fruits of *C. sativa* contained maximum quantity of flavonoid as compared to other plants tested. The aqueous extract of the flower of *C. sativa* possessed highest FRAP value followed by the flower of *A. mexicana* and the fruit of *C. procera*. The values of ratios of different polyphenolic compounds present in the plant extracts indicated that the flower of *D. metal* contained maximum flavonoids and minimum phenolics. These results suggested that the levels of total phenolics and flavonoids contents as well their FRAP indices varied not only from one plant to the other but also in their different parts tested. These results indicated that despite the presence of some toxic ingredients, these plants contained high antioxidant activity and sufficient quantity of flavonoids and phenolics in their varying parts, which may be exploited for certain medicinal or pharmacological formulations.

## Figures and Tables

**Table 1:** Total antioxidant activity of different plant preparations in terms of ferric reducing antioxidant power (FRAP).

S. number	Name of the plant	Plant part used	Extract type	FRAP (*μ*M Fe^++^ g^−1^)
1		Leaf		33.6 ± 0.14
2	*Argemone mexicana *	Stem		8.40 ± 0.14
3		Flower		69.1 ± 0.28
4		Fruit		11.8 ± 0.07
5		Leaf		24.7 ± 1.13
6	*Datura metal *	Stem		9.60 ± 0.00
7		Flower		21.3 ± 0.28
8		Fruit		23.1 ± 0.77
9		Leaf		38.7 ± 0.43
10	*Calotropis procera *	Stem	Aqueous	11.3 ± 0.77
11		Flower		24.5 ± 0.42
12		Fruit		41.3 ± 1.20
13		Leaf		15.8 ± 0.07
14	*Thevitia peruviana *	Stem		21.5 ± 0.70
15		Flower		19.2 ± 0.21
16		Fruit		24.2 ± 0.31
17		Leaf		34.0 ± 0.02
18	*Cannabis sativa *	Stem		14.5 ± 0.35
19		Flower		74.8 ± 1.93

The FRAP values for different parts of the plants *A. mexicana, D. metal, C. procera, T. peruviana *and* C. sativa *have been determined as described in Section 2. The values are the average of three independent experiments.

**Table 2:** Ratio of different polyphenolic compounds in the plant extracts (total flavonoids/total phenolics).

S. number	Name of the plant	Plant parts used	Extract type	Ratio of polyphenolic compounds (total flavonoids/total phenolics)
1	*Argemone mexicana *	Leaf		0.331
2		Stem		0.200
3		Flower		0.169
4		Fruit		0.324
5	*Datura metal *	Leaf		0.181
6		Stem		0.500
7		Flower		0.750
8		Fruit		0.251
9	*Calotropis procera *	Leaf		0.234
10		Stem	Aqueous	0.500
11		Flower		0.222
12		Fruit		0.209
13	*Thevitia peruviana *	Leaf		0.018
14		Stem		0.189
15		Flower		0.478
16		Fruit		0.253
17	*Cannabis sativa *	Leaf		0.155
18		Stem		0.260
19		Flower		0.129
